# Patient-reported long-term outcome following allogeneic hematopoietic stem cell transplantation in pediatric chronic myeloid leukemia

**DOI:** 10.3389/fonc.2022.963223

**Published:** 2022-09-29

**Authors:** Oliver Schleicher, Annkathrin Horndasch, Manuela Krumbholz, Stephanie Sembill, Claudia Bremensdorfer, Desiree Grabow, Friederike Erdmann, Axel Karow, Markus Metzler, Meinolf Suttorp

**Affiliations:** ^1^ Pediatric Hematology and Oncology, Department of Pediatrics and Adolescent Medicine, Comprehensive Cancer Center Erlangen-EMN (CCC ER-EMN), University Hospital Erlangen, Friedrich-Alexander University Erlangen-Nürnberg, Erlangen, Germany; ^2^ German Childhood Cancer Registry, Division of Childhood Cancer Epidemiology, Institute of Medical Biostatistics, Epidemiology and Informatics (IMBEI), University Medical Center of the Johannes Gutenberg University, Mainz, Germany; ^3^ Pediatric Hemato-Oncology, Medical Faculty Carl Gustav Carus, Technical University, Dresden, Germany

**Keywords:** pediatric CML, HSCT, long-term follow-up, health-related quality of life (HRQoL), Fact-BMT, participant self-reported outcome, cGVHD

## Abstract

**Background:**

Pediatric CML is very rare. Before the introduction of tyrosine kinase inhibitors (TKIs), allogeneic hematopoietic stem cell transplantation (HSCT) from a donor -if available- was the standard cure attempt. Data on the long-term outcome and health-related quality of life (HRQOL) in former pediatric CML patients undergoing HSCT are lacking.

**Study question:**

We investigated long-term survivors’ self-reporting to a questionnaire sent out to patients formerly enrolled in pediatric CML-HSCT trials.

**Methods:**

Individuals with CML transplanted at age <18 years were identified from the German Childhood Cancer Registry database. Long-term survivors received a questionnaire based on the SF-36 and FACT-BMT asking them to self-report HRQOL issues. (Ethical vote #541_20 B, Medical Faculty, University of Erlangen-Nürnberg).

**Results:**

111/171 (64.9%) individuals survived HSCT long-term and 86/111 (77.5%) fulfilled all inclusion criteria and received the questionnaire. 37/86 (43%) participants (24 female, 13 male, median age at HSCT 12 years [range 2-18], median age at the time of the survey 29 years [range 18-43]) responded after a median follow-up period of 19 years (range 4-27) after HSCT. 10/37 (27%) participants underwent no regular medical follow-up examinations. Self-reported symptoms like chronic graft-versus-host disease (cGvHD)-associated organ impairments and conditioning regimen consequences could causatively not sharply be separated in each case. Complains comprised hypothyroidism (N=11, 30%), infertility (N=9, 24%), lung problems, dry eyes (each N=7, 19%), skin alterations (N=6, 17%), hair problems (N=4, 11%), and sexual dysfunction (N=3, 9%). 10 (27%) participants experienced 13 CML relapses after a median interval from HSCT of 31 months (range 2-93). Only one patient underwent 2nd SCT after failure of relapse treatment with TKIs. Six secondary malignancies (dysplastic melanocytic nevus and ALL, basal cell carcinoma (N=2), rhabdomyosarcoma, and thyroid carcinoma developed in 5 (13%) participants. As assessed by the SF-36 questionnaire, impaired physical health was mainly associated with cGvHD. The mental component summary score showed that also participants without cGvHD scored significantly lower than the general population. When assessed by the FACT-BMT, participants with cGvHD scored significantly lower while participants without cGvHD scored even 5 points higher than the data from controls. 18 (49%) participants considered the sequelae of HSCT an obstacle to education. Out of the total cohort, N=20 (54%), N=7 (19%), N=5 (14%), and N=4 (11%) participants worked full time, part-time, were unemployed, or had not yet finalized their education, respectively. 20 (54%) participants lived as singles, 8 (22%) lived in a partnership, 6 (16%) were married, and 3 (8%) had been divorced. Four (11%) participants reported a total number of 7 children.

**Conclusion:**

This first assessment of HRQOL in former pediatric patients with CML surviving HSCT for more than two decades demonstrates self-reported satisfactory well-being only in the absence of cGvHD. Research-based on self-reported outcomes sheds light on former patients’ perspectives and provides an additional layer of valuable knowledge for pediatric and adult hematologists. Regular follow-up examinations are mandatory helping to avoid that late secondary neoplasias, CML-relapse, and disorders forming the broad range of possible long-term consequences of HSCT are not detected too late.

## Introduction

The introduction of targeted therapy for chronic myeloid leukemia (CML) by tyrosine kinase inhibitors (TKI) fundamentally changed the course of this disease. Following the availability of the first generation TKI imatinib and the second generation TKIs dasatinib and nilotinib for children and young adults, hematopoietic stem cell transplantation (HSCT) as curative treatment was postponed from an upfront approach recommended in the last millennium to a third line treatment option. This practice change for children and adolescents began soon after licensing imatinib for minors in November 2003 and covered a transition period during some of the following years (1). Results from a phase I study performed by the Children’s Oncology Group, including dosing and pharmacological details, were published in Nov. 2004 (2), while first experiences in children with CML generated in Germany and France were published two years later (3). Today, HSCT is still required for a small proportion of pediatric patients with CML in case of TKI failure or a primary diagnosis of CML in advanced phases.

HSCT is associated with considerable acute and chronic toxicity. This holds especially true for the scenario in the 1980ies - 1990ies when unrelated donor HSCT for children with CML had a fatality rate in the range of 30-40% (4–6). In pediatric survivors of HSCT, the occurrence of severe chronic graft versus host disease (cGvHD) in conjunction with its required immunosuppressive treatment is associated with adverse physical and psychological effects (7–9), disability (10), and poor quality of life (11–14). These significant morbidity problems arise in a particularly vulnerable developmental phase normally essential for a patient’s school education and professional training.

Most reports on pediatric patients with CML on follow-up after HSCT comprise small numbers due to the rarity of the disease in the first two decades of life and focus on survival and partially on cGvHD. Even in series comprising larger cohorts of pediatric patients transplanted over a prolonged period, quality of life has not been analyzed in detail (4, 15–18).

This lack of data is partly due to the fundamental structural barriers to transition from pediatric care to continued follow-up in adult hemato-oncology services unless structured transition programs are in place. Data protection concerns and changing the family name after marriage might also build additional hurdles for long-term follow-up studies. The German Childhood Cancer Registry records all CML cases from Germany diagnosed up to the age of 18 and obtains consent for continued follow-up contact (19). Since the reporting data from all national pediatric oncology treatment centers are compiled there and regularly synchronized with the data from the residents’ registration offices and civil registries, up-to-date contact data and survival status are available. This allows full tracking of childhood and adolescent cancer patients across age boundaries, and in addition, the representativeness of responders compared to the remaining registered individuals can be verified (20, 21).

The main objective of the study presented here was to investigate to what extent former pediatric patients treated for CML by allogeneic HSCT and surviving long-term exhibit as young adults a different health-related quality of life (HRQOL) profile compared with the general population and compared with patients transplanted for other malignancies. We decided to conduct this study as a participant-reported outcome (PRO) study. The HRQOL and symptom burden were assessed with a paper-based questionnaire including CML-specific items and the standardized SF-36 and FACT-BMT.

While multiple reports are published on the HRQOL of former HSCT patients with acute leukemias or benign hematological disease, the study presented here is the first to address the cohort of long-term survivors after HSCT for pediatric CML.

## Methods

Potential participants were identified using the German Childhood Cancer Registry maintained by the Division of Childhood Cancer Epidemiology at the Institute of Medical Biostatistics, Epidemiology and Informatics (IMBEI) from the University Medical Center of the Johannes Gutenberg University at Mainz, Germany. Eligibility criteria were: diagnosis of CML, treatment according to a registered pediatric CML trial, HSCT performed as part of the treatment more than five years ago and a confirmed mailing address. Eligible participants were sent a package containing a letter describing the study, informed consent forms, and a questionnaire to be completed and returned in a postage-paid envelope. Individuals who did not respond within two months were reminded by a second letter from the study team. No financial or other compensation was offered to the participants for completing the questionnaire. The study was approved by the University of Erlangen-Nürnberg Institutional Review Board (Ethical vote #541_20 B) and all participants provided written informed consent.

HRQOL was assessed using the Medical Outcomes Survey 36-item Short Form (SF-36) (22, 23). The current German version used in this study asks about perceived health and functioning over the past week and has been evaluated in adult survivors of childhood cancer (24, 25). The SF-36 contains eight scales, which are categorized into a physical health domain and a mental health domain (25, 26). The physical health domain consists of the physical functioning, role physical, bodily pain, and general health subgroups. The mental health domain is defined by the subgroups of vitality, social functioning, role emotional, and mental health. Each of the two domains of SF-36 yields a summary score: physical component summary (PCS) and mental component summary (MCS). Higher scores indicate better HRQOL. The SF-36 has been shown to be a valid and reliable measure with CML patients in multiple trials (22). Age-standardized norm data (N=576) of a representative population sample for Germany from 1994 (N=2914) were taken from the SF-36 questionnaire manual. The evaluation was also done using the validated SPSS syntax (26).

Transplant-specific HRQOL was assessed by the German version of the FACT-BMT questionnaire, version 4, which consists of the Functional Assessment of Cancer Therapy-General (FACT-G) (27 items) and the Bone Marrow Transplant Subscale (BMTS) (23 items). Transplant-related non-hematologic toxicities with long-term impact as assessed comprised cardiac, respiratory tract and lung, gastrointestinal, hepatobiliary, renal and urinary, bone and musculoskeletal, nervous system, ear and labyrinth, eye, endocrine, metabolism and nutritional, reproductive, and psychiatric disorders. Data on the occurrence of malignant neoplasms, relapse of CML, non-neutropenic infections, surgical and medical procedures, and social circumstances were retrieved.

The FACT-G evaluates the therapy-associated quality of life of cancer patients *via* self-reporting based on Likert scales (ranging from 0 to 4, where 0= “not at all,1= “a little bit”, 2= “somewhat”, 3= “quite a bit”, and 4= “very much”) in the dimensions of physical well-being, emotional well-being, functional well-being, and social well-being. By summing all five subgroups, the FACT-BMT total score can be calculated. The FACT is a validated (27) and frequently used test instrument to assess HRQOL in differing age groups and follow-up intervals (28, 29) and has been translated into several languages, including German (30). The license to use the questionnaire was granted by “FACIT.org” [Ponte Vedra, FL 32082-4161, USA, www.FACIT.org]. The evaluation was carried out according to the manual. The data collected was compared with a standardized and age-matched Austrian comparison cohort (31). The statistical tests included the Kolmogorov-Smirnov test (p<0.05) to identify collected variables that were not normally distributed. The comparison of the study population with the norm sample was performed using Welch’s t-test. Using the Mann-Whitney test, the two non-normally distributed and unrelated groups with and without cGvHD were tested for significant differences.

## Results

### Recruitment of long-term survivors

**Figure 1:**
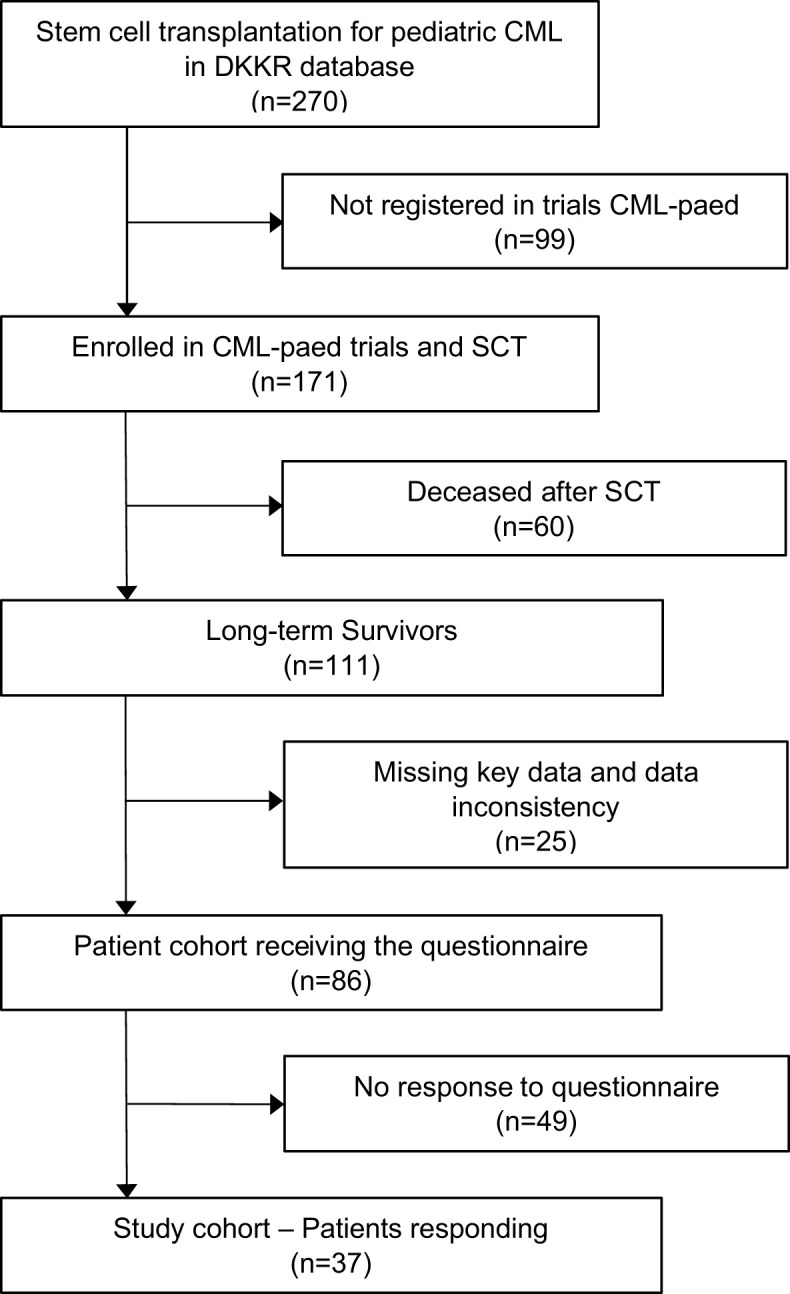
Consort diagram illustrating the recruitment of the participants into the survey.

The German Childhood Cancer Registry (DKKR) recorded 270 individuals diagnosed with CML in the age below 18 years who underwent HSCT. Of these, 171 were enrolled in clinical trials CML-paed I or CML-paed II (Ethical vote EK282 122 006, registered at EUDRACT-2007-001339-69 and Clinical-Trials.gov NCT00445822) and received a standardized treatment approach prior to HSCT between 1985 and 2016. Sixty patients had deceased after HSCT. Among the group of 111 long-term survivors, key data were missing in 25 individuals. The questionnaire was sent out to the remaining 86 individuals. Thirty-seven participants responded to the questionnaire resulting in a response rate of 43% (**Figure 1**).

**Figure 2:**
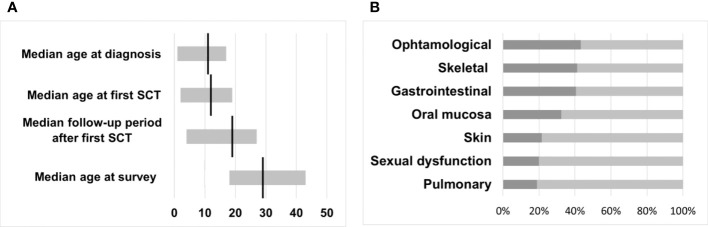
**(A)** Median time intervals with a range in years. **(B)** Transplant toxicity and percentage of participants reporting physical late-effects occurring after HSCT.

Twenty-four (65%) female and 13 (35%) male survivors responded to the questionnaire with a median age at the time of the survey of 29 years (range 18 - 43). CML was diagnosed between May 1993 and April 2013 in the responding cohort at a median age of 11 years (range 1 - 17). The interval from diagnosis to first HSCT ranged from 2 - 46 months (median 7), resulting in mean age at first transplantation of 12 years (range 2 - 19). The overall follow-up period after the first SCT of the cohort was 4 - 27 years, with a median of 19 years (**Figure 2**).

Seventeen pediatric patients (N=17/37, 46%) were transplanted before 2002 when imatinib was available only for adults enrolled in clinical trials. Fourteen out of the remaining 20 participants were still treated by the “classical” upfront HSCT approach without prior TKI administration from 2003 to 2007. The remaining six patients received prior TKI treatment for a median duration of 13 months (range 2-50) and, following current pediatric recommendations, underwent transplantation because of advanced phase at diagnosis and/or TKI treatment failure in the years 2008 - 2017 (32–34).

### General health condition and disease status

The general health status of transplanted patients results from genetic predispositions, lifestyle-related risk factors, therapy-related late effects, and diseases unrelated to the preceding CML and transplantation. Therefore, a definitive separation of individual diseases and transplant-related morbidity is not possible. To support a structured presentation of the health status and the avoidance of redundancies, we have grouped individual symptoms and diseases in the following text and as shown in **Figure 2**.

#### Body mass index and cardiovascular disorders

The median BMI of 35/37 individuals responding to this question was 23.0 kg/m^2^ (range 17.3 - 42.4). Twenty-two (63%) exhibited a BMI within the normal range (>18.5 - <25.0) following the NIH and WHO definition for white adults. 4/35 participants (11%, two females, two males) had underweight with a BMI <=18.5 kg/m^2^ and 5 participants (14%) were overweight with a BMI in the range 25.0 - 30.0 kg/m^2^ and 4/35 participants (11%) were obese with a BMI >30 kg/m^2^ (30.4, 31.6, 31.7, and 42.4, respectively).

While one female participant reported heart insufficiency with edema, none of the other 36 participants experienced heart or vascular problems like coronary artery disease (CAD) or a history of stroke, respectively. Two individuals reported irregular heartbeats without further specifications. Five out of 37 participants (13.5%) reported hypertension, which was observed only transiently over several years in two women while taking hormonal contraceptive medication. Three (8%) participants described vascular thrombosis. However, the underlying data could not establish an association with TKI therapy.

#### Endocrine and metabolic disorders

Hypothyroidism had developed in 11 participants (30%) of the cohort post-HSCT, including Hashimoto thyroiditis in two females and the development of thyroid nodules in another two participants respectively. Two female participants had diabetes mellitus (DM), one with type 1 DM diagnosed prior to the manifestation of CML and the other with a BMI of 31.6 kg/m^2^ insulin resistance had developed 18 years post HSCT at the age of 26 years. Two participants (5%) reported abnormalities in their cholesterol serum level and lipid metabolism without indicating further details. No participant reported abnormalities in the uric acid blood serum level.

#### Gastrointestinal disorders and renal disorders

Six of 37 participants (16%) reported gastric and bowel problems. One participant suffered from short bowel syndrome, and each one participant complained of diarrhea since HSCT and a mushy stool consistency because of so far undiagnosed reasons. Irritable bowel syndrome was reported by two participants, accompanied by lactose intolerance by one of them. The sixth participant claimed gastroesophageal reflux disease. Three participants reported elevated liver enzymes without further specification. No one has stated renal problems.

#### Neurological and psychological disorders

Two participants reported epilepsy, one of which was considered to be due to CNS mycosis following treatment for secondary acute leukemia. One participant experienced psychogenic seizures. Two participants were affected by frequently recurring migraines, and two other participants reported suffering from severe psychological distress. Vertigo was reported to occur regularly in 4/36 participants (11%) and frequently in one participant (3%). One participant reported pronounced symptoms of fatigue and impaired memory.

#### Medication and substance abuse

5/37 (13%) participants took sleeping pills only in exceptional circumstances, and in 1/37 (3%) participants, sleeping pills were prescribed for a prolonged period by a physician. 31/37 (84%) participants said they had not taken sleeping pills at any time. Similarly, tranquilizers were never taken by 29/37 (78%) participants. Tranquilizers were prescribed by a physician in 2/37 (5%) participants or taken without prescription by another participant (3%). 5/37 (13%) individuals took tranquilizers only under exceptional circumstances. 29/37 (78%) participants required analgesics, and only eight (22%) participants had never taken painkillers. In 7/37 (19%) participants analgesics were prescribed by a physician. 5/37 (13%) participants took painkillers without a prescription, and 17/37 (46%) took analgesics only under exceptional circumstances. One individual reported the use of appetite suppressants and stimulant agents without prescription.

23/37 (62%) participants denied drinking alcohol, smoking, or other substance abuse. Out of the remaining 14 (38%) participants, 12 individuals (32% of the total cohort) consumed alcohol one day per week (N=8), two days per week (N=3), or four days per week (N=1), respectively. Six (16%) participants were smokers (4 women, 2 men) and consumed a median of 52 (range 2 - 156) cigarette packages per year. Cannabis was consumed regularly every day by 1 participant beginning at the age of 24 (16 years after SCT) over the following three years, while another 25-year-old participant transplanted when 16 years old indicated to smoke cannabis only on single occasions with a frequency of every two months.

### Transplant-related morbidity and oncological aspects

#### Relapse of CML

Ten participants (3 males, 7 females) experienced one or more relapses at a median time interval from HSCT to the first relapse of 31 months (range 2-93). While in eight participants only one relapse was diagnosed, in each one participant two consecutive relapses at month 31 and month 72 and three consecutive relapses at month 30, month 37, and month 68 after HSCT, respectively, were observed, thus summing up to a total of 13 relapses in the cohort of 10 participants. No prophylactic TKI administration was performed post SCT in the whole cohort, however, when molecular relapse (defined as ratio BCR::ABL1/ABL1 >0.1% on the IS scale or a log fold increased ratio of BCR::ABL1/ABL1) was diagnosed *via* regular monitoring of minimal residual disease, TKI treatment was given preemptively. Three of the 13 relapses occurred in the first year after HSCT (at month 2, month 3, and month 8, respectively) and could be successfully managed by reducing the immunosuppression, a single donor lymphocyte infusion, and administration of ponatinib in the third case, respectively. Three participants relapsed late at 66, 83, and 93 months after HSCT, respectively, and could be successfully treated by TKIs. In total seven participants experiencing nine molecular relapses received TKI treatment. A single patient underwent a second HSCT when relapse was diagnosed 19 months after the first HSCT and was treated with imatinib and dasatinib for 7 months without achieving a major molecular response.

#### Secondary malignant neoplasia and family history of malignant diseases

Two participants developed basal cell carcinoma 13 and 15 years after HSCT. Another individual reported an unspecified malignant mole 3 years after HSCT and Ph-negative acute lymphoblastic leukemia as another hematological malignancy 7 years after HSCT. One participant developed thyroid carcinoma without indicating the interval post-HSCT; another was diagnosed with soft tissue sarcoma 15 years post-HSCT.

Blood cancers were diagnosed in relatives of 6/37 (16%) participants. In five cases, one of the grandparents was affected by CLL, plasmacytoma, lymphoma, and two unspecified hematological neoplasia at an advanced age. In the sixth case, an aunt from the father’s side was affected by not further specified blood cancer. Parents, siblings, and children were not affected in any of these cases. Non-hematological cancers were diagnosed in the relatives of 22/37 (62%) participants. In most cases, common cancer types (breast cancer, prostate cancer, colorectal cancer, lung cancer) at typical age were reported. In seven cases, the parents were affected by cancers. None of the participants had siblings affected by non-hematological cancers. Those participants who had children stated that none of their children was diagnosed with non-hematological cancers.

#### Transplant toxicity and physical late effects

##### a) Skin alterations associated with cGvHD

Eight (22%) participants suffered from skin cGvHD graded as extended in two individuals and limited in the remaining six participants. While one participant gave no specific information on complaints, skin dryness as the leading symptom was reported by all of the remaining seven participants, followed by erythema, disturbed pigmentation, sclerotic skin changes (each N=4 participants), and itching and sores or blisters (each N=3 participants). Three participants with cGvHD reported hair loss or thinning hair; however, conditioning with busulfan might also be claimed as a causative agent in these cases.

##### b) Oral mucosa, dental alterations, and transplant-related gastrointestinal complications

Oral and dental problems were indicated by 12 (32%) participants but further specified only by 11 participants. Gingivitis was the leading symptom reported by nine participants, followed by erythema (N=5), xerostomia (N=3), lichen (N=2), and frequent herpes eruptions in one participant. Four participants described dental caries with loss of teeth reported by one participant. Delayed teeth eruption and microdontia occurred in three participants transplanted at 2, 6, and 8 years, respectively, who all had received busulfan-based conditioning. Fifteen (40%) participants of the cohort complained of GI-associated problems during and after transplantation. 11 participants reported diarrhea. The remaining participants suffered from cGvHD of the GI tract and reported nausea (N=8) and vomiting (N=6). One participant reported dysphagia resulting from GvHD of the upper GI tract.

##### c) Ophthalmological disorders

Eye problems were indicated by 16 (43%) participants and further specified by 12 participants. Cataract was reported by 4 participants requiring lens replacement in two of them. TBI as part of the conditioning had been applied in three of these individuals, while the fourth participant had been treated with steroids for severe cGvHD. Ten participants reported dry eyes accompanied by redness in 5 of these participants and a diagnosis of keratoconjunctivitis sicca in 4 out of these participants. One participant reported herpes keratitis as a complication.

##### d) Skeletal system and muscle complaints

Three participants did not fill in this section of the questionnaire. Fourteen (41%) out of the remaining 34 participants complained of muscle and skeletal problems. Osteoporosis was the leading symptom (N=8), followed by painful muscle stiffness (N=6), joint contractures as a result of cGVHD (N=4), and arthrosis of larger joints (N=3). Five participants had experienced longitudinal growth impairment because of the conditioning (busulfan N=4, TBI N=1) and/or treatment of cGvHD by steroids.

##### e) Pulmonary late effects and infectious diseases

*S*even (19%) participants reported dyspnea as a complication of chronic lung GvHD. Bronchiolitis obliterans syndrome had developed in four of these seven participants in association with frequent respiratory tract infections and pneumonia. Four participants (11%) reported infections, including bacterial meningitis after SCT in one case.

##### f) Sexual function and fertility

34/37 (92%) participants reported no medical problems concerning the outer genital. One male reported reduced testicular volume diagnosed 1-2 years after SCT. He has received testosterone injections every 10 weeks since the 7th year post SCT. One female individual reported symptoms of a sicca syndrome for 8 years after SCT. Another patient reported unspecific symptoms. Sexual dysfunction was reported as rarely occurring by 4/35 (11%) participants (1 male, 3 females) and regularly occurring by 3/35 (9%) participants (1 male, 2 females). 28/35 (80%) participants reported no sexual dysfunction.

Infertility was reported by 15 (12 female, 3 male) out of 32 (47%) individuals who responded to this set of questions. Conditioning prior to SCT was based on TBI in eight of these participants and on busulfan in the remaining seven. Eight out of 15 participants provided more detailed information (5 female, 3 male). The male participants indicated hypogonadotropic hypogonadism 4 - 6 years after transplantation and infertility after irradiation. Female participants have reported irregular menstrual cycle, secondary amenorrhea, premature menopause, exhausted ovarian reserve, and infertility after irradiation. Out of the remaining 22 responders (12 females, 10 males), 17 individuals indicated not to be infertile without giving specific information or did not undergo further diagnostics, while the remaining 5 participants did not answer this question.

Four (11%) participants reported a total number of seven children. Three male participants (age at SCT 7, 16, 18 years, respectively, conditioning regimen applied Bus16/Cy120, TBI 14.2/Cytarabine150/Cy120, Bus16/Cy120/ATG60, respectively) each had fathered two children and one female participant (following conditioning with Bus16/Cy120/ATG60 at the age of 16 years) had given birth to one child after oocyte donation.

#### Organizational aspects of aftercare and satisfaction with medical care

**Figure 3:**
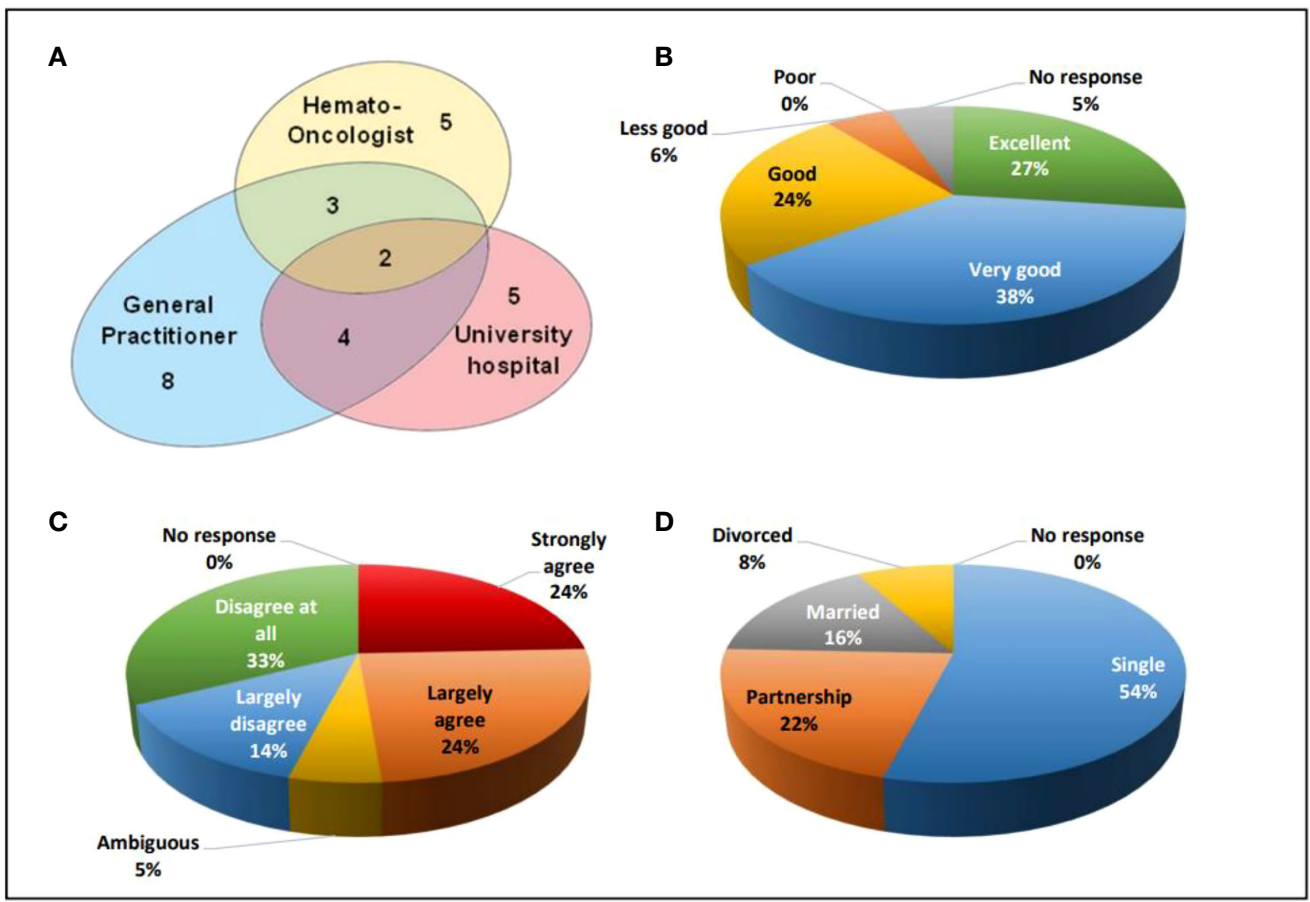
**(A)** Venn diagram indicating the organizational aspects of aftercare, **(B)** Satisfaction with medical care from 1 (excellent) to 5 (poor), **(C)** HSCT as an obstacle for career from 1(strongly agree) to 5 (disagree at all), **(D)** social status of the participants.

For regular follow-up post-SCT examinations, 27 participants (73%) visited a physician at least once a year. The remaining 10 (27%) participants did not at all participate in medical examinations at fixed intervals. 18 out of those 27 participants went to a single institution only, namely 8, 5, and 5 participants were examined by a general practitioner, by an hemato-oncologist in registered practice, or at the ambulance of a university hospital experienced in HSCT, respectively. Two participants regularly sought advice in parallel from a general practitioner, a hemato-oncologist, and from university hospital staff, while four participants consulted a general practitioner and a university hospital in parallel, and another three participants consulted a general practitioner and a hemato-oncologist in parallel (**Figure 3A**).

The frequency of medical visits in those 27 individuals participating in recommended follow-up examinations varied. While three (11%) participants did not indicate the intervals, another 3 (11%) participants, 11 (41%) participants, and 10 (37%) participants underwent medical examinations at 3-monthly, 6-monthly, and yearly intervals, respectively. Two out of 37 (5%) participants did not reply to the question on their satisfaction with medical aftercare. Out of the remaining participants, 10 (27%) individuals judged their medical care as excellent, 14 (38%) participants as very good, 9 (24%) participants as good, and 2 (6%) participants as less good (**Figure 3B**).

#### HSCT as an obstacle in school education/professional training

Being asked on a statement whether the procedure of HSCT and its sequalae had been an obstacle to their education at school and/or professional training and/or studying at the university, each 9 (24%) participants considered this “strongly agree” and “largely agree”. Two (5%) participants were ambiguous, while 5 (14%) participants and 12 (33%) participants stated that this was “largely disagree” and “disagree at all”, respectively (**Figure 3C**).

A delay in finalizing their education because of hospital stays was reported in total by 8 (22%) participants. Six (16%) participants had lost each 1 year, while 1 participant had lost 2 years, and another had lost 4 years, respectively. One (3%) participant had left school without graduating. Two (5%) participants graduated from junior high school (level C), 14 (38%) participants from secondary high school achieving level B, while the remaining 20 (54%) participants graduated at level A.

#### Social status and employment after HSCT

20 (54%) participants out of the total cohort were unmarried and lived as singles. 8 (22%) participants lived in a partnership, 6 (16%) participants were married, and 3 (8%) participants had been divorced (**Figure 3D**). At the time when filling in the questionnaire, 20 (56%) participants worked full time, 7 (19%) participants worked part-time, 5 (14%) participants were unemployed and looking for a job, 4 (11%) participants had not yet finalized their education and were studying while 1 participant did not reply to this question.

### Quality of life assessment

#### HRQOL of the participants compared to general norm data

In the physical health domain of the SF-36 questionnaire, reduced quality of life was found in the physical functioning score (Norm sample 95.11, all participants 83.65, participants without cGvHD 88.85, participants with cGvHD 71.36) and general health score (Norm sample 75.84, all participants 66.22, participants without cGvHD 72.19, participants with cGvHD 52.09) (**Table 1**). The scores of other subgroups and the physical component summary score did not differ significantly from the general population norm.

**Table 1:** Physical health as scored by the SF-36 questionnaire and level of significant differences in participants with and without GvHD. and in comparison to the norm sample.

SF-36 scales	A. Norm sample (n=576)	B. Transplated patients (n=37)	C. no cGvHD (n=26)	D.cGvHD (n=11)	Mean difference (95% Cl)	P value
**Physical health**						
** Physical functioning (PF)**	**95.11 (10.79)**	**63.65 (23.08)**	**88.85 (18.29)**	**71.36 (29.08)**		
A vs. B					11.46 ±3.821 (3.718 to 19.20)	0.0048**^a^
A vs. C					6.260 ±3.615 (-1.174 to 13.69)	0.0953^a^
A vs. D					23.75 ± 8.779 (4.202 to 43.30)	0.022*^a^
C vs. D						0.0136*^b^
**Role physical (RP)**	**90.75 (24.56)**	**89.19 (28.20)**	**94.23 (21.57)**	**77.27 (39.46)**		
A vs. B					1.560 ± 4.748 (-8.038 to 11.16)	0.7442^a^
A vs. C					-3.480 i 4.352 (-12.40 to 5.435)	0.4307^a^
A vs. D					13.48 ± 11.94 (-13.07 to 40.03)	0.2849^a^
C vs. D						0.0423*^b^
**Bodily pain (BP)**	**86.50 (22.49)**	**83.56 (23.58)**	**91.77 (16.41)**	**63.82 (26.95)**		
A vs. B					2.940 ±3.988 (-5.118 to 11.00)	0.4653^a^
A vs. C					-5.270±3.352 (-12.12 to 1.581)	0.1266^a^
A vs D					22.68 ± 8.180 (4.519 to 40.84)	0.0192*^a^
C vs. D						0.0011**^b^
**General health (GH)**	**75.84 (16.58)**	**66.22 (23.49)**	**72.19 (21.42)**	**52.09 (22.94)**		
A vs. B					9.620 ± 3.923 (1.681 to 17.56)	0.0189*^a^
A vs. C					3.650 ± 4.257 (-5.095 to 12.39)	0.399^a^
A vs. D					23.75 ± 8.155 (5.615 to 41.89)	0.0153*^a^
C vs. D						0.0167*^b^
**Physical component summary score (PCS)**	**54.35 (6.92)**	**52.25 (10.00)**	**55.37 (6.19)**	**44.89(13.39)**		
A vs. B					2.100 ± 1.669 (-1.273 to 5.478)	0.216^a^
A vs. C					-1.020 ± 1.248 (-3.576 to 1.536)	0.4206 ^a^
A vs. D					9.460 ± 4.043 (0.4539 to 18.47)	0.0413* ^a^
C vs. D						0.013*^b^

(SD), ^a^Welch’s t-test, ^b^Wilcoxon-Mann-Whitney test.

**Figure 4:**
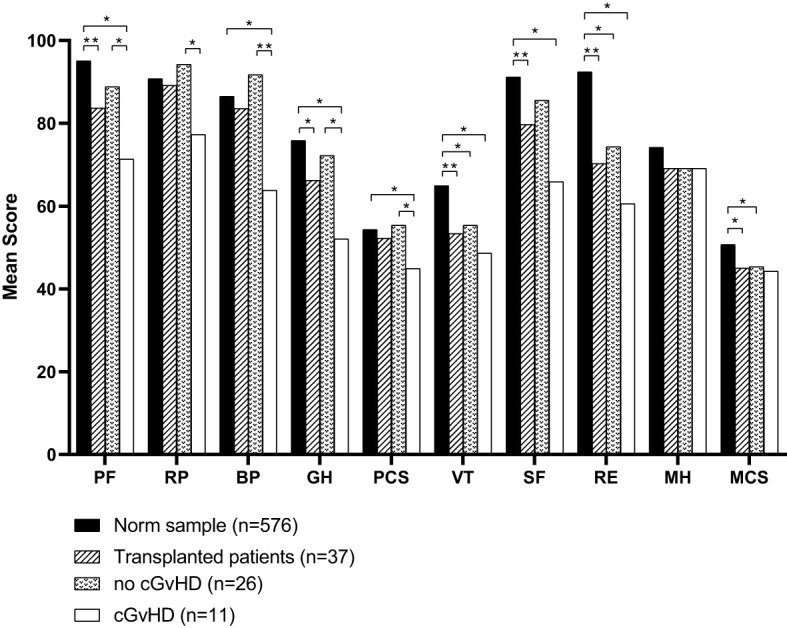
Mean scores of all domains assessed by the SF-36 questionnaire compared to the norm sample and separated into participants with or without cGvHD. For abbreviations, see the legend of **Figure 6**. Levels of significance are indicated by *denoting p<0.05 and **denoting p<0.01.

Compared to the age-standardized comparison population, the cohort in this survey showed a decrease in HRQOL in all mental health domains of the SF-36 questionnaire (**Table 2**). All subgroups showed a significant decrease, except for mental health. The median score of the mental component summary score was significantly lower (**Figure 4A**). The FACT-BMT questionnaire did not show significant differences between our survey group and the general population norm (**Table 3**).

**Table 2:** Mental health as scored by the SF-36 questionnaire and level of significant differences in participants with and without GvHD. and in comparison to the norm sample.

SF-36 scales	A. Norm sample (n=576)	B. Transplated patients (n=37)	C. no cGvHD (n=26)	D. cGvHD (n=11)	Mean difference (95% Cl)	P value
**Mental health**						
**Vitality (VT)**	**64.93 (17.00)**	**53.38 (20.95)**	**55.38 (20.78)**	**48.64 (21.57)**		
A vs. B					11.55 ± 3.516 (4.438 to 18.66)	0.0022**^a^
A vs. C					9.550 ±4.136 (1.056 to 18.04)	0.0290*^a^
A vs. D					16.29 ± 6.542 (1.759 to 30.82)	0.0315*^a^
C vs. D						0.4337^b^
**Social functioning (SF)**	**91.18 (16.38)**	**79.73 (23.63)**	**85.58 (18.27)**	**65.91 (29.63)**		
A vs. B					11.45 ± 3.944 (3.467 to 19.43)	0.0061** ^a^
A vs. C					5.600 ±3.647 (-1.386 to 13.09)	0.1364^a^
A vs. D					25.27 ± 8.960 (5.338 to 45.20)	0.018*^a^
C vs. D						0.0513 ^b^
**Role emotional functioning (RE)**	**92.43 (20.59)**	**70.27 (40_;_66)**	**74.36 (38.07)**	**60.61 (46.71)**		
A vs. B					22.16 ± 6.739 (3.507 to 35.81)	0.0022**^a^
A vs. C					18.07 ± 7.515 (2.612 to 33.53)	0.0237* ^a^
A vs. D					31.62 ±14.11 (0.4130 to 63.23)	0.0476* ^a^
C vs D						0.4903 ^b^
**Mental health (MH)**	**74.18 (15.51)**	**69.08 (15.88)**	**68.08 (16.57)**	**69.08 (14.87)**		
A vs. B					5.100 ± 2.689 (-0.3333 to 10.53)	0.0651 ^a^
A vs. C					5.100 ±3.313 (-1.698 to 11.90)	0.1354 ^a^
A vs D					5.090 ± 4.530 (-4.943 to 15.13)	0.2864 ^a^
C vs D						0.9929^b^
**Mental component summary score (MCS)**	**50.75 (7.81)**	**45.05 (11.19)**	**45.37 (11.34)**	**44.31 (11.33)**		
A vs. B					5.700 ± 1.863 (1.919 to 9.481)	0.0041** ^a^
A vs. C					5.376 ± 2.243 (0.7566 to 9.995)	0.0243* ^a^
A vs. D					6.440 ±3.432 (-1.188 to 14.07)	0.0895 ^a^
C vs. D					1.064 ± 4.076 (-7.470 to 9.598)	0.7613 ^b^

(SD), ^a^Welch’s t-test, ^b^Wilcoxon-Mann-Whitney test.

**Table 3:** Scores as reported by the FACT-BMT questionnaire and level of significant differences in participants with and without GvHD. and in comparison to the norm sample.

FACT-BMT scales	A. Norm sample (n=106)	B. Transplated patients (n=37)	C. no cGvHD (n=26)	D. cGvHD(n=11)	Mean difference* (95% Cl)	P value
**Phyical well-being (PWB)**	**25.74 (2.89)**	**24.24 (4.63)**	**25.58 (3.23)**	**21.09 (5.96)**		
A vs. B					1.460 ± 0.8116 (-0.1735 to 3.093)	0.0786^a^
A vs. C					0.1200 ±0.8933 (-1.287 to 1.527)	0.8636^a^
A vs. D					4.610 ± 1.819 (0.5830 to 8.637)	0.0287*^a^
C vs. D						0.0066**^b^
**Emotional well-being (EWB)**	**20.45 (3.89)**	**20 .72 (3.26)**	**21.46(3.01)**	**18.98 (3.17)**		
A vs. B					-0.2200 ± 0.6590 (-1.533 to 1.093)	0.7394^a^
A vs. C					-0.9600 ± 0.7014 (-2.370 to 0.4503)	0.1775^a^
A vs. D					1.520 ± 1.028 (-0.6951 to 3.735)	0.1625 ^a^
C vs. D						0.0164* ^b^
**Functinal well-being (FWB)**	22.35 (5.24)	22.69 (4.50)	23.95 (3.39)	19.73 (5.15)		
A vs. B					-0.3400 ± 0.8980 (-2.130 to 1.450)	0.7061 ^a^
A vs. C					-1.600 ± 0.8373 (-3.276 to 0.07592)	0.0609 ^a^
A vs. D					2.620+1.634 (-0.9323 to 6.172)	0.1343 ^a^
C vs. D					4.220 ± 1.689 (0.5927 to 7.847)	0.0306*^b^
**Social well-being (SWB)**	**22.22 (4.46)**	**23.49 (3.91)**	**23.61 (4.06)**	**23.21 (3.70)**		
A vs. B					-1.270 ± 0.7751 (-1.270 ± 0.7751)	0.1058 ^a^
A vs. C					-1.390 ± 0.9064 (-3.220 to 0.4404)	0.1328 ^a^
A vs. D					-0.9900 ±1.197 (-3.571 to 1.591)	0.4228 ^a^
C vs. D						0.616^b^
**FACT-G Total score**	**90.92 (12.33)**	**91.15 (13.07)**	**94.59 (13.07)**	**83.01 (14.11)**		
A vs. B					-0.2300 ± 2.460 (-5.151 to 4.691)	0.9258 ^a^
A vs. C					-3.670 ± 2.829 (-9.404 to 2.064)	0.2027 ^a^
A vs. D					7.910 ± 4.420 (-1.753 to 17.57)	0.0995 ^a^
C vs. D					11.58 ±4.967 (1.130 to 22.03)	0.0238* ^b^
**Bone marrow transplant subscale (BMTS)**		**31.42 (6.19)**	**32.88 (4.63)**	**27.97 (8.12)**		
C vs. D						0.0224* ^b^
**FACT-BMT Trail Outcome Index (TOI)**		**76.36 (13.74)**	**62.41 (10.02)**	**68.79 (16.78)**		
C vs. D						0.0272* ^b^
**FACT-BMT total score**		**122.57(18.57)**	**127.48 (15.24)**	**110.98(21.24)**		
C vs. D						0.0562 ^b^

(SD), ^a^Welch’s t-test, ^b^Wilcoxon-Mann-Whitney test.

#### Impact of cGvHD on reported HRQOL

In participants who did not report suffering from cGvHD, no significant difference in HRQOL from the general population norm was observed in all four sub-scores of the physical health domain and the physical summary component score. In the mental health domain decreased HRQOL was reported in vitality, role emotional functioning, and the mental component summary score. Also, no significant deviation in quality of life from the standardized comparison cohort was found in the FACT-BMT questionnaire. Overall, this cohort reported an increased median FACT-G total score.

**Figure 5:**
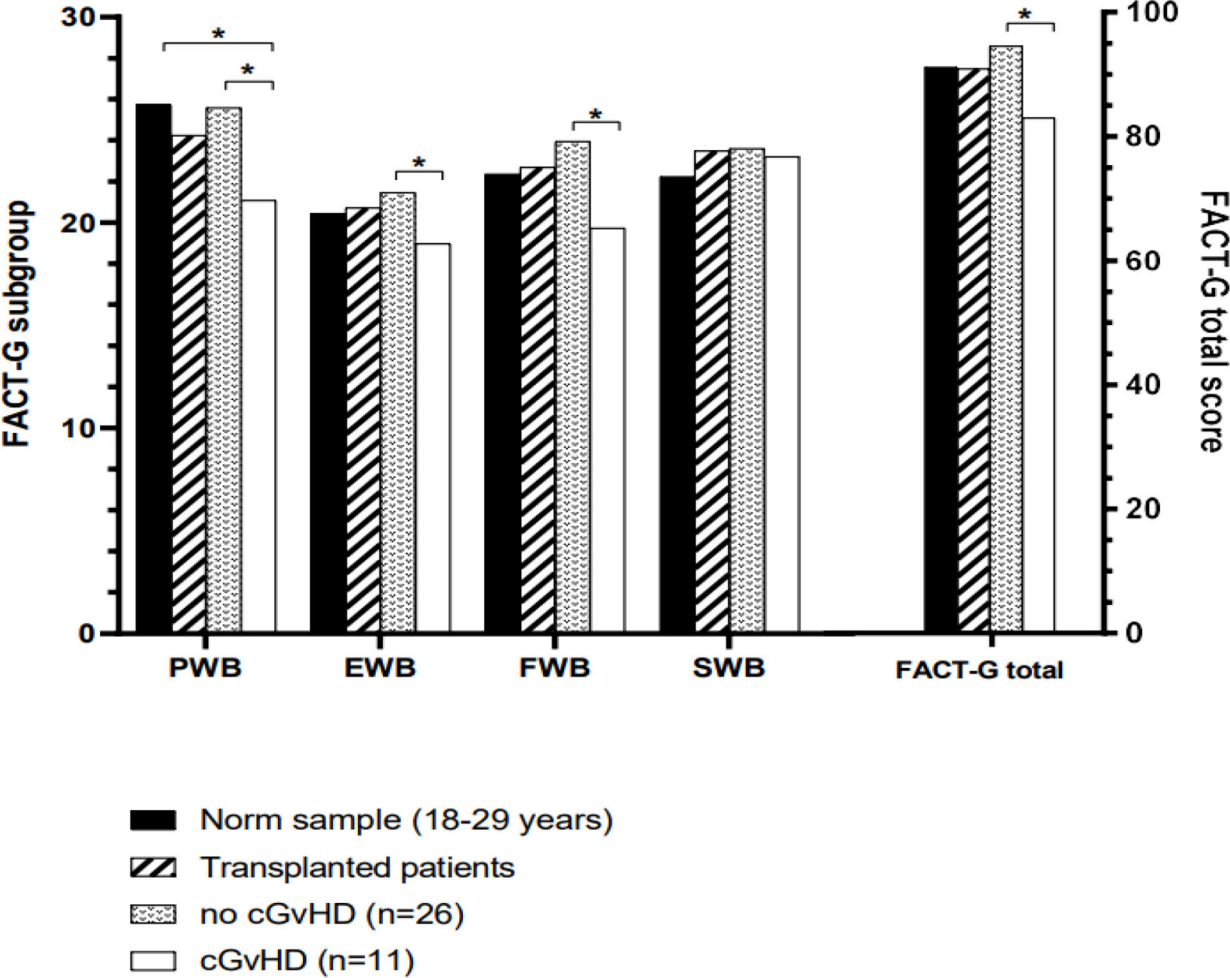
Mean scores of all domains assessed by the FACT-BMT questionnaire compared to the norm sample and separated into participants with or without cGvHD. For abbreviations, see the legend of **Figure 6**. The Level of significance is indicated by *denoting p<0.05.

Participants with cGvHD (N=11, 30%) showed lower median values compared to the standardized comparison cohort in all physical quality of life domains, with significant decreases in physical functioning, bodily pain, general health, and the physical component summary score. In the latter three categories, significantly reduced values in the physical domain were also reported compared to the group of participants without cGvHD (**Figures 4**, **5**).

**Figure 6:**
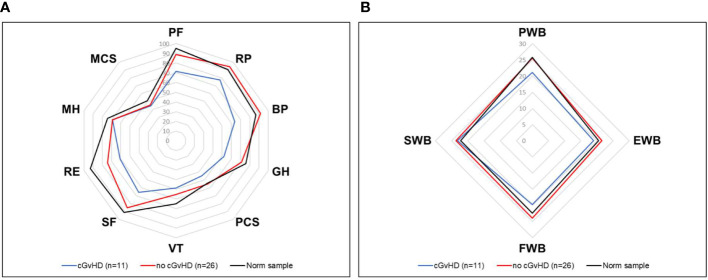
HRQOL of generalized norm cohort (n=576, black line) and transplanted pediatric CML patients with (n=11, blue line) and without (n=26, red line) cGvHD. **(A)** The SF-36 questionnaire includes the physical health (PF), role physical (RP), bodily pain (BP), general health (GH), physical component summary score (PCS), vitality (VT), social functioning (SF), role emotional functioning (RE), mental health (MH) and mental component summary score (MCS) subscales. **(B)** The FACT-BMT questionnaire consists of the 4 main dimensions physical well-being (PWB), emotional well-being (EWB), functional well-being (FWB), and social well-being (SWB).

Participants with self-reported cGvHD had lower mean scores in all mental health categories, including the mental component summary score (MCS) compared to the standardized comparison cohort. In comparison to the cohort of participants without reported cGvHD, quality of life was decreased in all subgroups except mental health, with no significant difference found between the respective MCS (**Figure 6A**). In the FACT-BMT questionnaire, participants with cGvHD had decreased mean scores in all subgroups, except social well-being, and FACT-G total score compared to the standardized comparison cohort. Compared to participants without reported cGVHD, the same groups showed significantly reduced mean scores (**Table 2**, **Figures 5**, **6B**).

#### Individual statements of long-term survivors

In the field for individual comments, the categorical entries of previous items were explained in more detail in some cases. However, in addition to these specific explanations, certain items were noted with special emphasis. The recurring comments related in particular to the need for more psychological support in the period around and after transplantation, the impairment caused by cGvHD, the lack of fertility protection options at the time of transplantation, and the psychological consequences of infertility.

## Discussion

CML is considered a stem cell disease, and in the pre-TKI era, HSCT could be offered as the only therapeutic approach promising a long-term cure. In those days, HSCT was recommended to be performed early after diagnosis of CML for patients in CP. However, the median age of CML at diagnosis of >60 years excluded most of the patients from HSCT. CML in the first two decades of life is very rare. As children and teenagers tolerate the transplant-associated toxicity better than adults, upfront HSCT within 6 months or 12 months after diagnosis for patients with an HLA-matched sibling or unrelated donor, respectively, was evaluated in the CML-paed-I trial between 1995 and 2004 (6). Especially in children, this resulted in a considerable proportion of HSCT recipients becoming long-term survivors.

This study aimed to identify cross-sectionally in former pediatric patients the post-transplantation characteristics of their health status associated with perception of quality of life, their educational level qualification, professional integration, and marital status after HSCT. Self-reported data were compared to literature on normal controls and patients transplanted for other malignancies. Most long-term follow-up studies in pediatric patients investigated the long-term impact of specific modalities of the conditioning regimen (e.g., TBI, reduced intensity conditioning) or the function of specific organs (e.g., gonads, left heart ventricular function, hypertension, longitudinal growth). HRQOL has been investigated in pediatric AML and ALL survivors but not in pediatric CML yet (35–38).

### Participation and response to the questionnaire and gender distribution

A response rate of 43% is in the normal range for long-time follow-up and HRQOL assessment by questionnaires. However, the gender distribution (65% females, 35% males) of the 37 responding participants is inverse to the typical distribution in pediatric CML at diagnosis or HSCT, with a male preponderance in the range of 54% to 65% males (6, 15, 17, 18, 39–41). Already in the cohort receiving the questionnaire (N=86), the gender distribution was skewed to female individuals (f=46 (54%), m=40 (46%)), and in the responding participants (N=37), the female proportion increased further. Whether females than males respond more often to (health-related) questionnaires is discussed controversially (42–45). Thus, the reasons why more females than males responded to the questionnaire in this trial remains speculative.

### Age at diagnosis and time intervals from diagnosis to filling in the questionnaire

The mean age at diagnosis was 11 years (range 1-17) which is in line with large cohorts of patients with pediatric CML. Self-evidently the upper age limit when collecting data for comparison on pediatric adolescents (14 vs. 16 vs. 18 vs. 20 years) heavily impacts the median age as the incidence of CML continuously increases in the second decade of life (39, 46).

Regarding a poorer outcome, if HSCT is performed late after diagnosis of CML, recommendations in the pre-TKI era clearly stated that in young patients, HSCT should be performed preferably in CML-CP and within the first year after diagnosis of CML (47–49). With the median interval from diagnosis to first HSCT of 7 months (range 2-46), this criterion was fulfilled by most of the responding participants.

After HSCT, the questionnaire was completed after a median time of 19 years (range 4-27). That is higher or comparable to the highest range of follow-up studies so far performed in children transplanted for mostly malignant diseases (35, 38, 50–54).

### Body mass index in transplanted pediatric patients

In a trial analyzing the longitudinal trends in BMI and outcomes of children (N=297) undergoing HSCT, a total of 40% of children were overweight and/or obese prior to and post SCT, but the proportion of obese subjects increased from 17% prior HSCT to 24% post-HSCT (55). In that study, there was a significantly larger BMI increase in patients conditioned with busulfan than those who received TBI. By contrast, another trial demonstrated that BMI declines significantly after allogeneic HSCT for childhood hematologic malignancies (N=179), primarily due to a substantial decrease in lean body mass (56). Also, in a French study on a smaller cohort of children with leukemia (N=49), the BMI post-HSCT was normal but decreased in boys having received TBI (57).

The cohort of children with CML enrolled in this trial differs from children with other malignancies as the participants received no polychemotherapy but only hydroxyurea prior to HSCT. In children with sickle cell anemia, chronic hydroxyurea treatment had no adverse effect on weight gain (58). The 14% and 11% proportion of participants in this study exhibiting overweight and obesity, respectively, is approximately only half the size of the general age-matched German population (59). However, the small number of participants must be considered.

### Hypertension and other cardiac and vascular risk factors

13.5% of the participants reported hypertension which is in line with other studies describing high blood pressure in long-term survivors of HSCT performed at pediatric age with a prevalence of 15% (60). In a cohort aged 18-39 years at follow-up, well comparable to our cohort (18-43 years), hypertension was observed twice as frequently as in the normal US population (53). Only one (3%) female participant reported heart insufficiency with edema in the cohort investigated here, but the increased incidence in individuals undergoing HSCT at pediatric age is a strong argument for regular aftercare. Besides obesity and high blood pressure, further risk factors for coronary artery disease comprise smoking, diabetes, lack of exercise, high blood cholesterol, depression, and excessive alcohol consumption (61–63). Smoking was denied by 84% of the participants, which is higher than the 70% to 75% proportion of non-smokers in the German population (64). 68% of the participants denied drinking alcohol which is considerably higher than the 13% proportion of adult non-drinkers in the German population (65).

### Diabetes mellitus

DM type 2 was diagnosed in one female participant at 36 years (18 years after SCT) and associated with obesity (BMI 31.6). That represents a rather low proportion (1/37 = 2.7%) in our trial. While the size of our cohort is comparable to most other pediatric reports on DM post-HSCT, a higher incidence is generally observed in other pediatric follow-up studies in the range of 5.6% (66), 5.8% (67), 16,8% (68), and 23% (69), respectively. An exceptionally large pediatric study with a median of 11 years of follow-up stems from Seattle and describes 38/748 (5.1%) patients who developed type 2 DM 1 to 24 years after HSCT, between 11 and 41 years of age (70). These pediatric results are much identical to data from adults (N=599) exhibiting DM post-HSCT in 6.5% of patients, mostly associated with cGvHD treatment and/or obesity (71). While a small cohort size generally may bias any interpretation of reasons for developing DM type 2 (e.g., type of conditioning, steroid treatment), participants requiring long-term immunosuppression must be considered at increased risk (72). Without a doubt, the existing pediatric data underline that evaluation of metabolic syndrome and glucose tolerance should be part of hormonal follow-up, which should be routinely proposed to all patients transplanted for CML (73, 74).

### Thyroid function

Thyroid abnormalities such as (subclinical) hypothyroidism, antithyroglobulin, and thyroid peroxidase antibodies, (euthyroid) Hashimoto thyroiditis, and increased frequency of thyroid cancer as long-term sequela is not restricted to TBI-based conditioning regimen only and affect HSCT survivors with a total frequency in the range of 20% - 35% (75–79). In a large trial from Seattle (N=791 children), multivariate analysis showed that thyroid dysfunction was more likely if patients were less than 10 years of age at transplant, but there was no difference between receiving total body irradiation or busulfan-based regimens (80).

Also, thyroid disorders might be observed in non-transplanted children with CML treated with TKIs (81). While thyroid dysfunction is relatively common after HSCT, it is more likely to occur in patients receiving prolonged immunosuppression for cGVHD; however, thyroid dysfunction does not appear to be related to an antibody-mediated autoimmune process (76). In the cohort described here, each two patients developed Hashimoto thyroiditis and thyroid nodules out of 30% of participants with hypothyroidism. That is well within the ranges reported for HSCT performed because of other underlying diseases, and no CML-specific findings or conditioning-specific associations were observed. The high 30% incidence of hypothyroidism strongly underlines that those children receiving HSCT should be monitored for thyroid abnormalities throughout life.

### Chronic Graft-versus-host disease and conditioning regimen-related toxicities

Among approximately 50-70% of adult and 20-50% of pediatric survivors of HSCT, cGvHD is observed (82–84). cGvHD symptoms and treatment are often associated with significant morbidity and decreased functional status and quality of life (11, 85, 86). Although some observers reported that the HRQOL is steadily rising with an increasing interval from HSCT, improvement and disappearance of these complications may take many years (87). Organ impairments may also persist permanently and, even as outlined in a report from a Scandinavian pediatric study, are likely to increase during extended follow-up, particularly in patients who have received TBI (88).

In the study presented here, 26 (70%) participants stated that they were free from cGvHD. This proportion is in line with observations in pediatric cohorts treated by HSCT for other malignancies. However, as the reported details resulted from self-assessment, specific problems such as gingivitis or xerostomia, dry eyes, or pulmonary late effects might be a consequence of either the conditioning regimen applied (TBI-based in 8/37 (22%) participants of the total cohort) or a result of donor cell alloreactivity (cGvHD in 11/37 (30%) participants) (89). Dyspnea was reported in 7/11 participants also exhibiting other features of cGvHD. A reduced pulmonary function has been attributed to both TBI-based and busulfan-based conditioning (90, 91).

### Relapse of CML

Data from the pre-TKI era, when transplantation represented the classical indication of treatment, demonstrated in large cohorts of adult patients that relapse is observed in patients transplanted in CP with a frequency in the range of 20% (92, 93). That also holds true for pediatric cohorts transplanted without prior TKI treatment (6, 15–17). A relapse rate of 27%, as reported by 10/37 participants in this study, is higher than what has been reported, but evidently, the small cohort size must be considered. As of note, the relapse rate seems to increase with time, as in patients younger than 29 years reported by a study from the CIBMTR, the cumulative incidence of relapse for the entire cohort (N=399) was 11% at 1 year, 18% at 3 years, and 21% at 5 years after HSCT (18). In the cohort reported here, 1^st^ relapse occurred even later in two participants at 5.5 years and 6.9 years after HSCT, respectively. Very late relapses of CML have been described in adult patients up to 25 years after HSCT and thus demonstrated the necessity for life-long monitoring of minimal residual disease (MRD) (94).

This cohort’s median time from HSCT to relapse was 34 months (range 2-83). Three out of the 13 relapses reported by the 10 participants occurred in the first year after HSCT (at month 2, month 3, and month 8) and could be successfully managed by reduction of the immunosuppression, a single donor lymphocyte infusion (DLI), and administration of ponatinib in the third case, respectively. As of note, only a single participant underwent a second HSCT after relapse was diagnosed at month 19 after HSCT. All remaining relapses were successfully managed with TKIs and/or DLI as salvage treatment.

Since 2000 a standardized approach to detect leukemic relapse by quantitative RT-PCR has been applied in the CML-paed trials. This tool detected jeopardizing relapses early on the molecular MRD level and resulted in preemptive TKI administration thus avoiding hematological relapse. Because in this cohort, only one relapse occurred before 2000, this approach might have contributed significantly to the success of early intervention with TKIs.

### Secondary neoplasias following HSCT

In this cohort of 37 participants, 6 secondary neoplasias were diagnosed in 5 (13%) individuals (3 males, 2 females) with a median latency of 13 years (range 4-16) after diagnosis of CML and median 13 years (range 3 - 15) after HSCT. Three participants reported skin cancers (two with basal cell carcinomas, one with malignant mole), and each one participant developed thyroid carcinoma, soft tissue sarcoma, and Ph-negative acute lymphoblastic leukemia (following the diagnosis of malignant mole 3 years before), respectively. TBI was administered to 3 of the 5 participants as part of the conditioning regimen prior to HSCT.

Other studies from larger pediatric HSCT cohorts (N=426, 90% receiving TBI) demonstrated an incidence of 4.7% of secondary solid tumors diagnosed after a median latency of 11.7 years (range 5.4-21.5), with thyroid carcinoma being the most frequent (9 out of 20) solid cancers (78). In an analysis by the Center for International Blood and Marrow Transplant Research registry (CIBMTR, 1995-2012) of 6028 pediatric patients transplanted for non-malignant diseases, secondary malignancies developed in 1.2% of the cohort; however, the median follow-up interval was shorter comprising only 7.8 years (95). In a Spanish study 19 cases of secondary malignancies were observed in a cohort of 371 former pediatric HSCT patients, with a cumulative incidence of 6%, 12%, and 36% at 15, 20, and 30 years of follow-up, respectively (96). Our data match these reports and highlight that the early diagnosis of secondary malignancies is one of the key tasks of long-life multidisciplinary post-transplant care.

### Follow-up at specialized centers after HSCT

Two decades ago, 60/171 (35%) pediatric patients enrolled in the CML-paed trials deceased following HSCT (see **Figure 1**). Also, those patients who had overcome early complications and survived long-term after HSCT are at a higher risk of late effects than the general population as discussed above. The need for long-term follow-up was early recognized and guidelines by a collaboration of international working groups, including pediatric patients, have been proposed (97–102). Nevertheless, many HSCT recipients who judged by self-estimation that they had no late effects and took no medications regularly often terminated their follow-up examinations at HSCT centers. In a study from Seattle, low adherence to follow-up was associated with male sex, lower physical functioning, the absence of cGvHD, a longer time after HSCT, poor knowledge of recommended tests, and concerns regarding medical costs (103). While the latter plays no role in Germany because of the social insurance system established, the constantly increasing frequency of HSCT associated with transplantation at older adult age and improved outcomes resulted in limited resources for aftercare at most HSCT centers and may have considerably contributed to a delegation of follow-up visits to oncologists or general physicians in private praxis. In a report from Japan, the cumulative incidence of follow-up termination at HSCT centers was 28% at 10 years and increased to 67% at 25 years after allogeneic HSCT (104). In another report from the IBMTR, including 3865 children, the 10-year cumulative incidence of being lost to follow-up amounted to 25% in pediatric allogeneic HSCT survivors (105). Our observation, that at a median FU of 19 years, 10 (27%) individuals did not participate in FU-examinations at all and only 5 (13%) participants are examined annually in a university hospital experienced in HSCT, sadly confirms these data.

Individuals followed at private practices may encounter several challenges as only well-trained expert physicians have sufficient knowledge about late effects or complicated and/or multiple comorbidities in HSCT survivors (51, 106, 107). Notwithstanding the data that the majority of participants (62%) in this trial highly acknowledged their medical aftercare as outstanding or very good, it might be difficult for a general physician to provide adequate diagnosis and treatment if complications of HSCT occur late after patients had terminated follow-up at their HSCT center. Thus, most importantly, communication in a network between transplant centers and all involved healthcare providers is of outstanding importance concerning unique HSCT survivorship issues (108, 109).

### School education, social status, and employment after HSCT

18 participants (48.6%) considered it “very true” or “true to a great extend” that CML and the HSCT procedure had been an obstacle to their education (**Figure 3C**), resulting in a delay of 1 to 4 years in graduation for 8 participants (21.6%). However, finally all but one participant graduated and the 54% proportion achieving the highest levels (“Fachabitur”, “Abitur”, Baccalaureate) qualifying for studying at a university of applied science (Fachhochschule) or university, respectively, is higher than in the age-matched general German population (110). Our data match a French study describing a median delay of 1.32 years until the final graduation at year 13 of school education was achieved in academics who underwent HSCT during childhood (N=59) (111). However, the number of students who received their secondary school diploma (Baccalaureate) was similar to the expected rate in the general French population for girls only (observed/expected=1.02) but significantly decreased for boys (observed/expected=0.48). The high proportion of female participants in our trial excluded such a comparison.

In a German log-term follow-up study on children with acute leukemia who did not undergo HSCT, survivors graduated at higher levels of school compared to the general population, with females 48.6% versus 38.0% and males 52.6% versus 35.8%, respectively (112). The established German system for support of pediatric patients with malignancies comprising individual lessons by a schoolteacher during stays in the hospital and at home, might have played an important role in achieving graduation at high levels (113).

In the cohort analyzed, graduation resulted in an 84% employment ratio (N=20 full-time, N=7 part-time) for those participants who had completed their education (N=32). An unemployment rate of 11% (N=4) out of all participants answering the questionnaire is higher than what is reported for the general German population, with a declining rate from 11.7% in the year 2005 to 5.7% in the year 2021 (114). Internationally, in a large cohort of 2844 young adult HSCT survivors (ages 18-32 years) who underwent HSCT at childhood age in the US between 1985 and 2010, an unemployment status was found age dependently in the range of 13-15% (115). In Japan, the unemployment rate in 107 survivors (age 18 - 24 years) of HSCT (at age 7-13 years) was found to be 17% (116). Although the unemployment rates are grossly in the same range, efforts to reintegrate unemployed individuals and supporting activities may differ nationally and thus make comparison difficult. However, in adults undergoing HSCT, a much higher rate of unemployment, early retirement, and inability to work in the range of 40 - 50% is observed, resulting in lower financial income compared to the time pre HSCT (117–119). As of note, in a small cohort of 18 long-term survivors of childhood lymphoblastic malignancies from Sweden, the findings indicated that unemployment or sick leave are associated with a decline in HRQOL rather than HSCT, cranial radiation therapy, present age, or sex (120).

### Gonadal dysfunction and parenthood

Infertility is a common sequela after pediatric HSCT if myeloablative conditioning is applied. However, the individual risk is influenced by the treatment prior to HSCT, the age at conditioning and differs for male or female sex (121). Individual rare parenthood cases have been described after both TBI-based or busulfan-based full ablative conditioning regimens (122–125). In a cohort of males from Scandinavia (N=98), all patients with TBI had azoospermia, while in patients treated with chemotherapy only, a higher cumulative cyclophosphamide equivalent dose was associated with an increased risk of azoospermia (54). In a very large European analysis, 62 988 pediatric patients received the first HSCT in EBMT centers between 1995 and 2016 and pregnancy was reported in 406 (0.6%) patients (126). Regarding conception modality, 13/25 (52%) of females conditioned with TBI and 50/52 (96%) of those conditioned without TBI conceived naturally. Male patients who had been conditioned with TBI achieved fatherhood but required assisted fertilization or used their cryopreserved sperm. In the last two decades, fertility preservation measures have been increasingly offered to patients prior to conditioning and consensus statements on this issue have been published (127, 128). Whether or not such measures had been offered to the participants in the cohort described here was not explicitly evaluated; however, the high ratio of 4/37 (11%) participants (3 male, 1 female) with children makes such an assumption highly probable.

### Health-related quality of life

The questionnaires SF-36, FACT-Leu, EORTC QLQ-CML24, and MDASI CML all have been proven to generate reliable data and are commonly applied to individuals with CML (129). Impairment of physical health, as reported by the participants of this trial and assessed by the SF-36 questionnaire, was mainly associated with cGvHD. Participants without cGvHD even exhibited a higher physical component summary score (PCS) than controls (see **Table Supplementary**); however, if cGvHD was present, the PCS score was significantly lower. Concerning mental health, the mental component summery score (MCS) showed that also participants without cGvHD scored significantly lower than the general population (see **Figure 4**). Thus, it may be concluded that cGvHD exhibits a negative impact on general health while HSCT in general causes a decline in the MCS score. Assessment based on the FACT-BMT detected no differences between the cohort investigated here and controls. However, when separated according to reported cGvHD, in analogy to data of the SF-36, participants with cGvHD scored significantly lower while participants without cGvHD scored even 5 points higher than controls.

These data match with findings in 75 Irish adult patients with CML (age 35 years at HSCT, range 14-55) undergoing sibling donor HSCT when assessed after a median 8 years post HSCT with the EORTC QLQ-C30 and showed no difference for scores in the physical, emotional and social domains or the overall Global Health Status/QoL (130). In contrast, a cohort of 29 pediatric patients transplanted in the UK for acute leukemia at a median follow-up of 5 years (range 1-14) after HSCT exhibited significant morbidity and compromised HRQOL when compared with the Pediatric Quality of Life questionnaire (PedsQL™ 4.0) to 29 non-transplanted patients (131). Also, in a single center analysis by the SF-36 from Padua/Italy performed 17 years (range 8-32) post HSCT on 21 pediatric patients, lower scores were reported on scales referring to bodily pain, general health, and physical and social functioning (132). In line with our data, in a Danish study comprising N=132 long-term survivors (>10 years) of HSCT performed at pediatric and <25 years of age, overall scores of HRQOL were similar to norm data (133).

### Weakness and strength of this analysis

We recognize the limitations of the findings as described in this report. In this retrospective analysis on a small cohort comprising 37 participants, data were not collected at an identical time point post HSCT. While earlier reports had shown no impact of the myeloablative conditioning regimen applied (Busulfan versus TBI) on overall survival (6), for statistical reasons HRQOL in defined very small sub-cohorts (e.g. participants conditioned with TBI (N=9), participants receiving pre-TKI treatment (N=6)) could not be compared to the remaining majority of the participants. Self-reported HRQOL may lack medically proven details, especially in those participants not involved in regular follow-up assessments. The long latency from HSCT to the time when the questionnaire was answered by the majority represented a challenge, as stated by some participants. Also, a small minority of the total cohort did not answer some items of the questionnaire. Possible confounding variables such as household income and resulting socio-economic status were not covered by the questionnaire.

The strength of this analysis lies in assessing a unique cohort of pediatric CML patients undergoing HSCT that has not been described so far. The median 19 years (range 4-27) interval from HSCT to answering the questionnaire is the longest so far reported and the amount of data accumulated enables pediatricians to take a glimpse at adult survivors of CML treated by HSCT. Instead of focusing on the frequency of selected aspects of HSCT sequelae as reported in other papers on follow-up after HSCT, a broad spectrum of HRQOL is described and analyzed. Last but not least, the positive response from many participants makes clear that families would benefit from counseling about possible long-term consequences of HSCT in CML.

## Conclusions

In an era of individual-centered medicine, research-oriented on the participant-reported outcome (PRO) sheds light on former patients’ perspectives and thus provides an additional layer of valuable knowledge for both pediatric and adult hematologists. Using PROs exhibits participants’ preferences and may ultimately improve communication with physicians. Adherence to regular follow-up examinations remains a cornerstone to avoiding that late secondary neoplasias (13% in this cohort), relapse of CML (27% in this cohort), and disorders forming the broad range of possible long-term consequences of HSCT (in this cohort: hypothyroidism 30%, infertility 24%, lung problems 19%, dry eyes 19%, skin alterations 17%, hair problems 11%, sexual dysfunction 9%) are not detected or made a subject of discussion too late. Participants suffering from cGvHD (30% in this cohort) reported a negative impact on their general quality of health, while participants without cGvHD exhibited no differences compared to controls. This first study on former pediatric patients with CML surviving for a long-time after HSCT provides new disease-specific data as a rational basis for disease management, hopefully resulting in improved overall HRQOL.

## Data availability statement

The raw data supporting the conclusions of this article will be made available by the authors, without undue reservation.

## Ethics statement

The study was approved by the University of Erlangen-Nürnberg Institutional Review Board (Ethical vote #541_20 B) and all participants provided written informed consent. The patients/participants provided their written informed consent to participate in this study.

## Author contributions

OS, MM, MS conceived the study and developed the design. CB, FE, AK, DG, AH, MK, OS, SS contributed to the acquisition and preparation of data. AK and OS conducted the statistical data analysis. OS, MM, MS wrote the first draft of the manuscript. All authors contributed to the data interpretation, critically reviewed the manuscript for important intellectual content, and revised the manuscript. All authors contributed to the article and approved the submitted version.

## Conflict of interest

The authors declare that the research was conducted in the absence of any commercial or financial relationships that could be construed as a potential conflict of interest.

## Publisher’s note

All claims expressed in this article are solely those of the authors and do not necessarily represent those of their affiliated organizations, or those of the publisher, the editors and the reviewers. Any product that may be evaluated in this article, or claim that may be made by its manufacturer, is not guaranteed or endorsed by the publisher.

## Glossary

**Table:**

Allo	Allogeneic
BMI	Body mass index
BMT	Bone marrow transplantation
BMTS	Bone Marrow Transplant Subscale
BP	bodily pain
CAD	Coronary artery disease
cGvHD	Chronic graft versus host disease
CML	Chronic myeloid leukemia
CP	Chronic phase
DKKR	Deutsches Kinderkrebs-Register
DM	Diabetes mellitus
EWB	emotional well-being;
FACT-G	Functional Assessment of Cancer Therapy-General
FWB	functional well-being
GH	general health
HRQOL	Health-related quality of life
HSCT	Hematopoietic stem cell transplantation
IMBEI	Institute of Medical Biostatistics, Epidemiology and Informatics
MCS	Mental component summary
MH	mental health
mo	month
NIH	National institute of health
PCS	physical component summary
PF	physical functioning
PRO	Participant reported outcome
PWB	physical well-being
RE	role emotional
RP	role physical
SCT	Stem cell transplantation
SF	social functioning
SWB	social well-being
TBI	Total body irradiation
TKI	Tyrosine kinase inhibitor
VT	vitality
WHO	World health organization
