# Corrigendum: Patient-reported long-term outcome following allogeneic hematopoietic stem cell transplantation in pediatric chronic myeloid leukemia

**DOI:** 10.3389/fonc.2022.1116757

**Published:** 2022-12-15

**Authors:** Oliver Schleicher, Annkathrin Horndasch, Manuela Krumbholz, Stephanie Sembill, Claudia Bremensdorfer, Desiree Grabow, Friederike Erdmann, Axel Karow, Markus Metzler, Meinolf Suttorp

**Affiliations:** ^1^ Pediatric Hematology and Oncology, Department of Pediatrics and Adolescent Medicine, Comprehensive Cancer Center Erlangen-EMN (CCC ER-EMN), University Hospital Erlangen, Friedrich-Alexander University Erlangen-Nürnberg, Erlangen, Germany; ^2^ German Childhood Cancer Registry, Division of Childhood Cancer Epidemiology, Institute of Medical Biostatistics, Epidemiology and Informatics (IMBEI), University Medical Center of the Johannes Gutenberg University, Mainz, Germany; ^3^ Pediatric Hemato-Oncology, Medical Faculty Carl Gustav Carus, Technical University, Dresden, Germany

**Keywords:** pediatric CML, HSCT, long-term follow-up, health-related quality of life (HRQoL), Fact-BMT, participant self-reported outcome, cGVHD

**Text Correction**

In the published article, there was an omission in the Acknowledgements section. The classification as part of the doctoral thesis of O. Schleicher was not integrated into the published article.

A correction has been made to **Acknowledgments**. The paragraph previously stated:

“The continuous financial support in data collection by “Sonnenstrahl e.V. Dresden - Förderkreis für krebskranke Kinder und Jugendliche” (Dresden, Germany), “Schornsteinfeger helfen krebskranken Kindern e.V.” (Dörfles-Esbach, Germany), and the “Madeleine-Schickedanz Kinderkrebsstiftung” (Fürth, Germany) is highly acknowledged.”

The full corrected Acknowledgments appear below:

**Acknowledgments**

The continuous financial support in data collection by “Sonnenstrahl e.V. Dresden - Förderkreis für krebskranke Kinder und Jugendliche” (Dresden, Germany), “Schornsteinfeger helfen krebskranken Kindern e.V.” (Dörfles-Esbach, Germany), and the “Madeleine-Schickedanz Kinderkrebsstiftung” (Fürth, Germany) is highly acknowledged. The present work was performed by O. Schleicher in fulfillment of the requirements for obtaining the degree “Dr. med.” at Friedrich-Alexander-Universität Erlangen-Nürnberg (FAU), Germany.

The authors apologize for this error and state that this does not change the scientific conclusions of the article in any way. The original article has been updated.

## Publisher’s note

All claims expressed in this article are solely those of the authors and do not necessarily represent those of their affiliated organizations, or those of the publisher, the editors and the reviewers. Any product that may be evaluated in this article, or claim that may be made by its manufacturer, is not guaranteed or endorsed by the publisher.

