# Supplementary material for: Patient-reported long-term outcome following allogeneic hematopoietic stem cell transplantation in pediatric chronic myeloid leukemia
Source: Front Oncol. 2022 Sep 29;12:963223. doi: 10.3389/fonc.2022.963223 (PMC9580018; doi:10.3389/fonc.2022.963223)
Supplement: Supplementary file 1 [file DataSheet_1.pdf]

Supplementary Materials

Questionnaire (english translation).. 2

1. General anamnesis

**1.1. Height**	cm	**1.2. Weight**	kg
**1.3.** Date of **CML diagnosis**	D D M M Y Y	**1.4.** Date of **transplantation**	D D M M Y Y

1.5. Do you currently have or have you had the following health complaints?

	yes	no	If yes, since when?	If yes, specify
**1.5.1. Cardiovascular diseases**				
a. Coronary heart disease / myocardial infarction	1	2		
b. Cardiac arrhythmias	1	2		
c. Heart failure / cardiac insufficiency	1	2		
d. Stroke	1	2		
e. Hypertension / high blood pressure	1	2		/ mmHg
f. Circulatory disorders	1	2		
**1.5.2. Metabolic diseases**				
a. Diabetes mellitus	1	2		☐ Typ I ☐ Typ II
b. Thyroid disorders	1	2		☐ Hypothyroidism<br>☐ Others:
c. Lipid metabolism disorders / cholesterol	1	2		
d. Uric acid metabolism disorders / gout	1	2		
e. Liver diseases	1	2		
f. Stomach / intestinal diseases	1	2		
g. Kidney diseases	1	2		☐ Requiring dialysis<br>☐ Others:
**1.5.3. Other diseases / complaints**				
a. Blood clotting disorders	1	2		
b. Infectious diseases	1	2		
c. Lung diseases	1	2		☐ COPD<br>☐ Asthma<br>☐ Others:
d. Seizure disorders / neurological diseases	1	2		☐ Epilepsy<br>☐ Others:
e. Autoimmune diseases	1	2		☐ Rheumatoid arthritis<br>☐ Hashimoto thyroiditis<br>☐ M. Crohn/ Colitis ulcerosa<br>☐ Others:
f. Infertility	1	2		
g. Others:	1	2		

2. Medical history with focus on transplantation and CML.

2.1. From when and how often did you experience the following complaints after transplantation (Tx)?
Please only indicate complaints that occurred for the first time after transplantation and specify any additional symptoms.

	Often	Sometimes	Seldom	Never	How long after transplantation?
a. Fever	1	2	3	4	
b. Genital infections	1	2	3	4	
c. Sexual dysfunction	1	2	3	4	
d. Dizziness	1	2	3	4	
e. Hearing disorders	1	2	3	4	
f. Dry eyes	1	2	3	4	
g. Skin irritations / rashes / redness	1	2	3	4	
h. Body hair disorders	1	2	3	4	
i. Others:	1	2	3	4	

2.2. Please mark all diseases / symptoms that occurred after the transplantation (Tx) by marking the corresponding suggestions in the first column. Please indicate how long after the transplantation (Tx) the symptoms first appeared and add more to the respective subgroup if necessary.

	yes	no	If yes, how long after Tx?	If yes, please specify:		
**a. Do you suffer from chronic graft-versus-host disease (cGvHD)?** What is the severity of cGvHD?	1	2		mild<br>1	moderate<br>2	severe<br>3
**b. Skin** For example: scarring, wrinkling, disorders of pigmentation, erythema, redness, itching, rash, dryness, lichen / nodules	1	2				
**c. Oral mucosa** For example: redness / erythema, lichen / nodules, dryness, gingivitis, caries, tooth loss, disturbed development of the teeth	1	2				
**d. Skeletal system** For example: Osteoporosis, muscle stiffness, muscle pain, tendonitis, soft tissue infections, growth disorders	1	2				
**e. Eyes** For examample: conjunctivitis, eye redness, dryness, keratoconjunctivitis sicca / dry eye, cataract	1	2				
**f. External genital** For example: Scarring, open areas, pain, fissures, redness, itching, dryness, lichen / nodules.	1	2				
**g. Digestive tract** For example: Dysphagia, nausea, vomiting, diarrhea, fatty liver, cirrhosis of the liver, bile stasis / cholestasis	1	2				
**h. Lung** For example: Shortness of breath, irritable cough, respiratory tract infections, pneumonia, bronchiolitis obliterans syndrome (BOS) / scarring of the lungs	1	2				

**2.3.** Have you experienced any other malignant diseases?<br>If yes, please specify.	☐ yes	☐ no

	yes	no	If yes, when?	If yes, please specify
a. Diseases of the blood	1	2		☐ Leukemia / blood cancer<br>☐ Myelodysplastic syndrome (MDS)<br>☐ Others:
b. Tumor diseases of the skin or mucous membrane	1	2		☐ Melanoma<br>☐ Others:
c. Other tumor diseases	1	2		☐ Nervous system / brain<br>☐ Thyroid gland<br>☐ Liver<br>☐ Others:

**2.4.** Have you had a relapse of chronic myeloid leukemia (CML)?<br>If yes, please specify.	☐ Yes, on: D D M M Y Y<br>☐ no

	yes	no	If yes, since when?	If yes, please specify
a. Treatment of relapse with tyrosine kinase inhibitors (TKIs)	1	2		☐ Imatinib<br>☐ Dasatinib<br>☐ Nilotinib<br>☐ Bosutinib<br>☐ Ponatinib<br>☐ Others:
b. Treatment of relapse with donor lymphocyte infusion (DLI)	1	2		How often?
c. Repeated stem cell transplantation (SCT)	1	2	D D M M Y Y	Place of transplantation

**2.5.** Do you regularly present to a physician for post-transplant care?<br>If yes, please specify.	☐ yes	☐ no

	yes	no	If yes, how many times per year?	Name of clinic / practice / doctor
a. General practitioner	1	2		
b. Outpatient specialist / oncologist	1	2		
c. Regional hospital	1	2		
d. University Hospital	1	2		
e. Transplant Clinic	1	2		

3. Extended medical history

**3.1.** Did malignant diseases of the hematopoietic system / blood cancer occur in your family / in blood relatives?	☐ yes	☐ no

	**yes**	**no**	**m** (maternal) **p** (paternal)	**If yes, specify:**
a. Parents	1	2		
b. Grandparents	1	2		
c. Uncle / aunt	1	2		
d. Siblings	1	2	-	
e. Children (biological)	1	2	-	

**3.2.** Have there been any other malignancies / cancers in your family / blood relatives?	☐ yes	☐ no

	**yes**	**no**	**m** (maternal) **p** (paternal)	**If yes, specify:**
a. Parents	1	2		
b. Grandparents	1	2		
c. Uncle / aunt	1	2		
d. Siblings	1	2	-	
e. Children (biological)	1	2	-	

3.3. Do you regularly take medication from the following groups?
Please indicate how often and under what circumstances the medication was taken.

	**Never taken**	**Prescribed by the doctor**	**Without prescription**	**Only in exceptional cases**
a. Sleeping pills	1	2	3	4
b. Sedatives	1	2	3	4
c. Painkillers	1	2	3	4
d. Appetite suppressants/ laxatives	1	2	3	4
e. Stimulants	1	2	3	4

3.4. Do you regularly consume one or more of the following substances? Please mark the appropriate suggestions and indicate how often and how much you consume per week.
Please specify any additional substances that are not mentioned below.

	**yes**	**no**	**Since when?**	**If yes, how often / much per week?**
a. Alcohol	1	2		Consumption on _____ days
b. Nicotine	1	2		_______ packs of cigarettes
c. Drugs<br>For example: Cannabis, LSD, cocaine, opiates, i.v. drugs	1	2		Consumption on _____ days
d. Other addictive substances:	1	2		Consumption on _____ days

**3.5.**	Please answer the following questions about your current vaccination status.<br>* If you are unsure, please consult the current STIKO vaccination calendar. You are also welcome to enclose a copy of your vaccination certificate.

	yes	no	If yes, specify:
a. Have you ever been vaccinated before?	1	2	-
b. Did you receive full basic immunization* as a child?	1	2	-
c. Do you have your vaccinations refreshed regularly?	1	2	-
d. Have you received any additional vaccinations*?	1	2	
e. Have you received any other vaccinations* after transplantation?	1	2	☐ Influenza<br>☐ Hepatitis A<br>☐ Travel vaccinations<br>☐ Others:
f. Do you think that sufficient vaccination protection is particularly important for you?	1	2	-

**3.6.**	Do you have any known allergies or intolerances to certain substances? Please mark the corresponding suggestions in the first column and specify additional allergies or intolerances if not mentioned below.

	yes	no	Others/ which ones?	(Since) when?
**a. Do you suffer or have you suffered from ....**<br>For example: Eye discomfort, nasal itching, sneezing, irritable cough, snoring, shortness of breath, skin reactions, gastrointestinal discomfort.	1	2		
**b. Are there any intolerances / allergies against**<br>For example: Medication, pollen, dust mites, animal dander, metals, food, cosmetics, occupational substances, insect bites	1	2		
**c.** Have new intolerances / allergies occurred or increased after transplantation?	1	2		

**3.7.**	Please mark with a cross where applicable and complete further details if necessary.

a. How would you rank your current medical care?	excellent	very good	good	less good	poor
	1	2	3	4	5
b. The transplant / its treatment have affected me in my professional or educational training.	strongly agree	largely agree	don't know	largely disagree	disagree at all
	1	2	3	4	5
c. Highest school degree (multiple answers possible)	Without general school leaving certificate	Graduation at level C	Gruaduation at level B	Graduation at level A	Delay of graduation by _____ years
	1	2	3	4	5
d. Current employment status (multiple answers possible)	Full-time	Part-time	Self-employed	Job-seeking	Student
	1	2	3	4	5
e. Current marital status / children (multiple answers possible)	unmarried	married	divorced	Living in a stable partnership	____ children
	1	2	3	4	5
